# Supplementary material for: Interaction of the pioneer transcription factor GATA3 with nucleosomes
Source: Nat Commun. 2020 Aug 18;11:4136. doi: 10.1038/s41467-020-17959-y (PMC7434886; doi:10.1038/s41467-020-17959-y)
Supplement: Supplementary file 1 — Supplementary Information [file 41467_2020_17959_MOESM1_ESM.pdf]

Supplementary Information

Interaction of the pioneer transcription factor GATA3 with nucleosomes

Hiroki Tanaka, Yoshimasa Takizawa, Motoki Takaku, Daiki Kato, Yusuke Kumagawa, Sara A. Grimm, Paul A. Wade, and Hitoshi Kurumizaka

Supplementary Figure 1

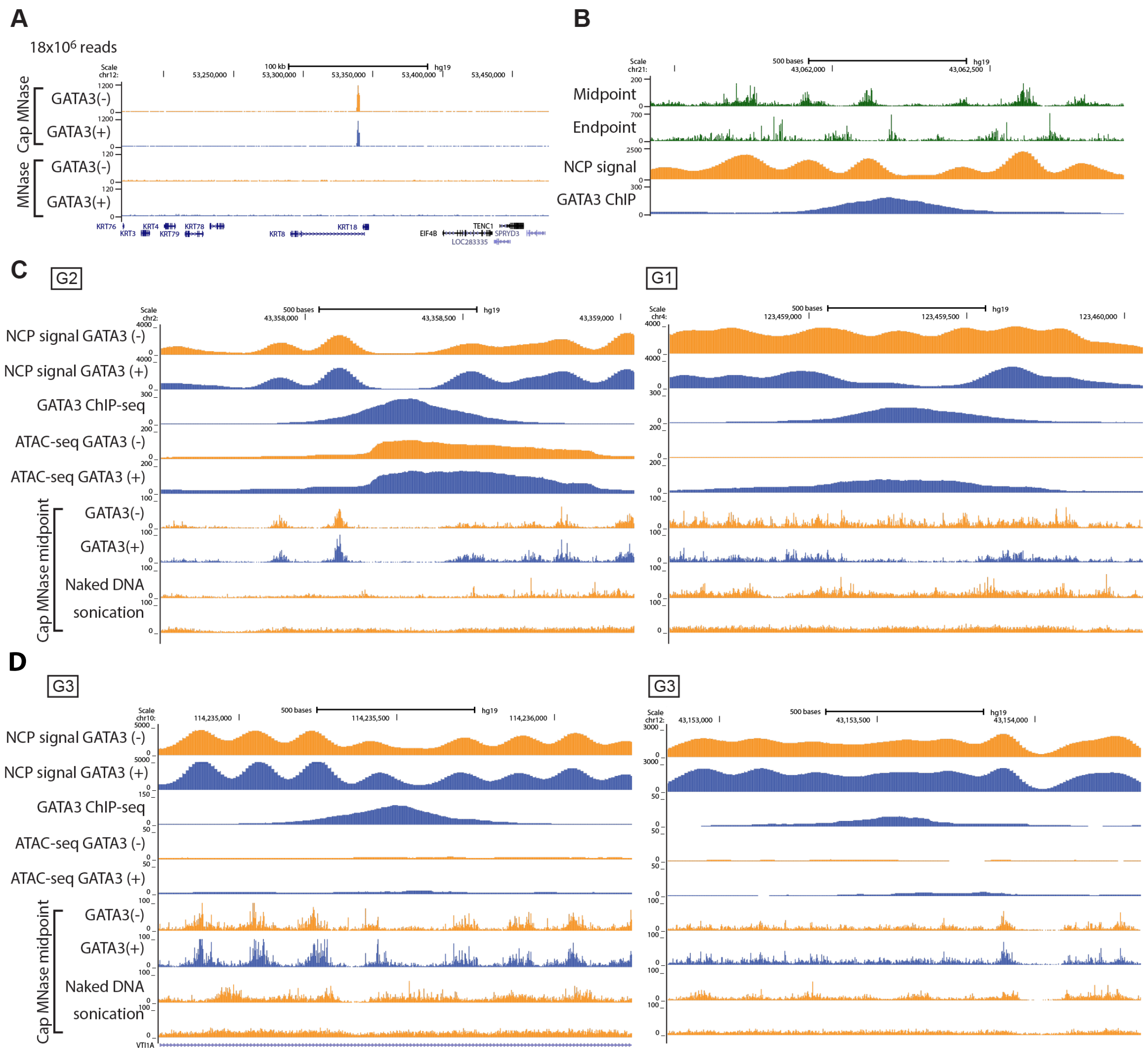

Supplementary Figure 1 Capture MNase-seq provides deep nucleosome mapping at individual GATA3 binding loci. **a,** Comparison between conventional MNase-seq and capture MNase-seq. Browser tracks show genome coverage from each MNase-seq method. **b,** Example of capture MNase-seq data at a GATA3 binding site. Midpoint or endpoint indicates sequenced nucleosomal fragment center (considered as dyad) or end (considered as naked DNA regions). Smoothed nucleosome signals (NCP signal) were calculated by iNPS [16]. **c,** Examples of nucleosome pattern in each GATA3 peak group. A subset of GATA3 binding loci show well-defined nucleosome positions, while others show non-uniform nucleosome positioning. **d,** Nucleosome positions at the G1 binding locus used in Figure 3c *in vitro* assay.

Supplementary Figure 2

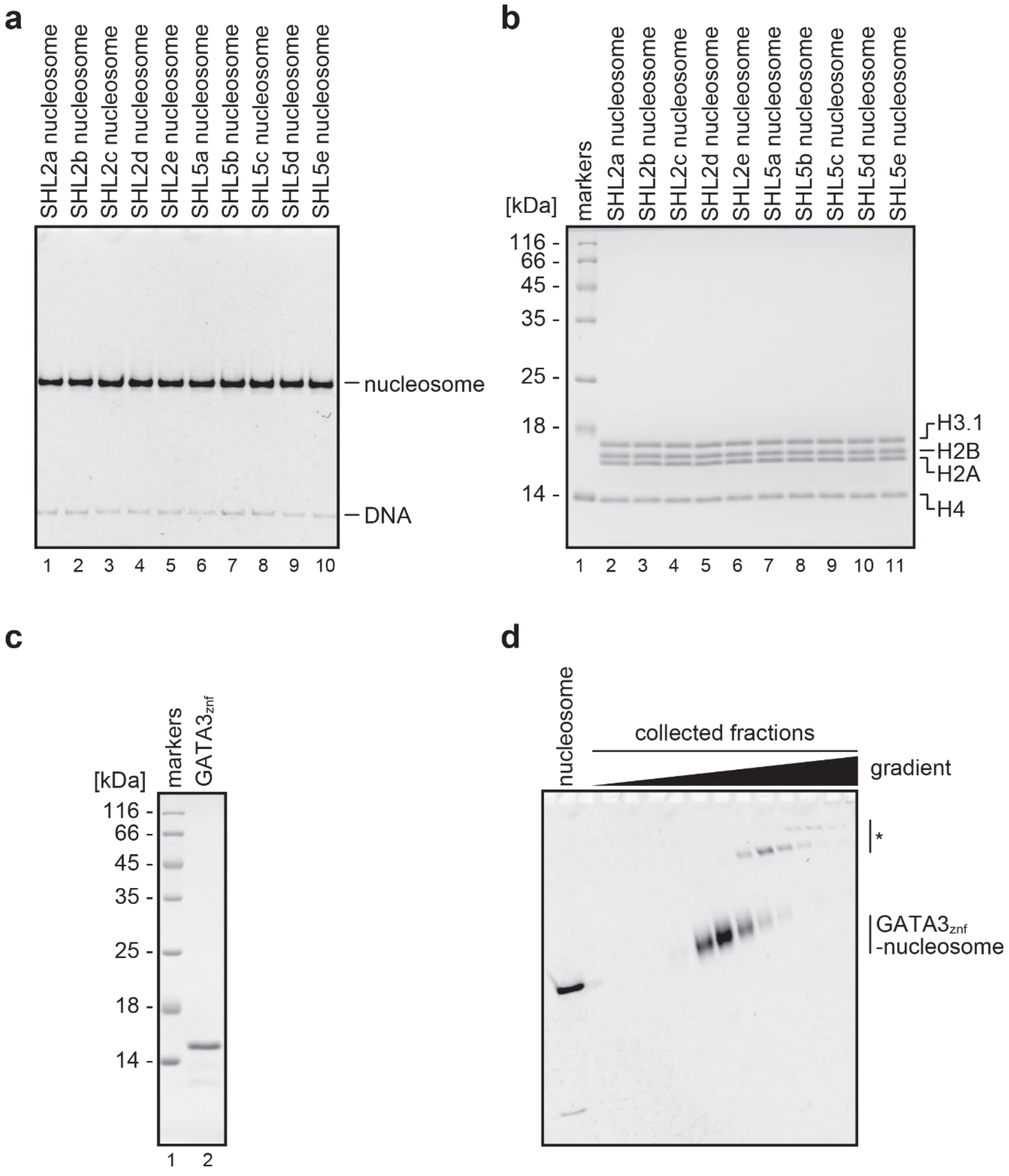

Supplementary Figure 2 Preparation of the nucleosomes, GATA3$_{znf}$, and the GATA3$_{znf}$-nucleosome complex.

a, Purified nucleosomes were analyzed by native-PAGE with ethidium bromide staining. A representative image is shown, this experiment was repeated 3 times with similar results. **b,** The histone contents of the purified nucleosomes were analyzed by SDS-PAGE with Coomassie Brilliant Blue staining. A

representative image is shown, this experiment was repeated 3 times with similar results. **c,** Purified GATA3$_{znf}$ was analyzed by SDS-PAGE with Coomassie Brilliant Blue staining. A representative image is shown, this experiment was repeated 3 times with similar results. **d,** GATA3$_{znf}$-nucleosome complex was purified by GraFix method, and the collected fractions were analyzed by native-PAGE with ethidium bromide staining. The vertical bar with an asterisk indicates nucleosome aggregates. A representative image is shown, this experiment was repeated 3 times with similar results.

Supplementary Figure 3

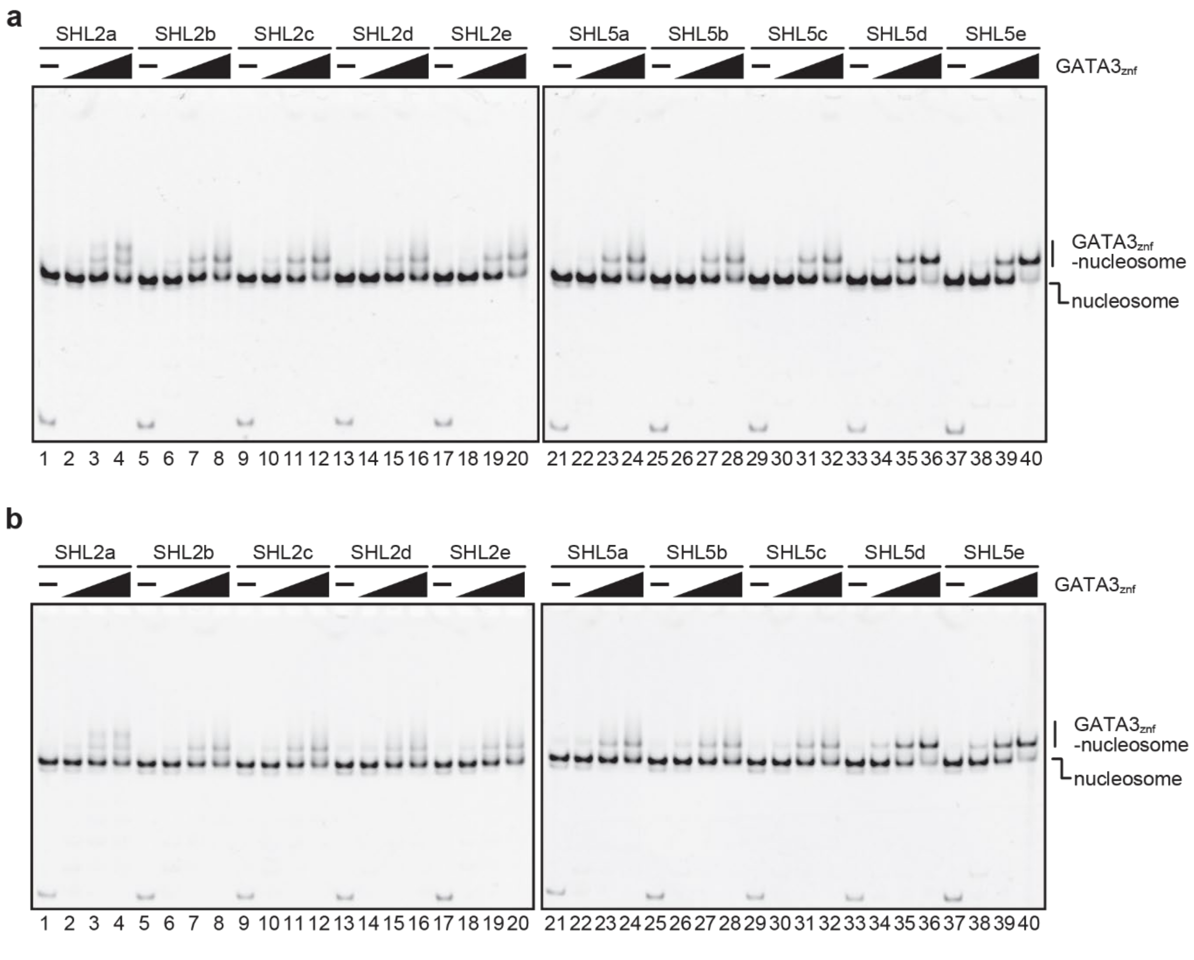

Supplementary Figure 3 Replication of GATA3 nucleosome binding
a-b. Each panel shows an independent biological replicate of the GATA3 nucleosome binding experiment depicted in Figure 2b. The experiment was performed with three independent biological replicates depicted in Figure 2b, Supplementary Figure 3a, and Supplementary Figure 3b. The nucleosome (0.33 μM) was mixed with GATA3$_{znf}$ (0 μM, 0.67 μM, 1.0 μM, and 1.33 μM). The reaction mixtures were incubated at 25°C for 30 min and were then analyzed by native-PAGE. The gel was stained with ethidium bromide.

Supplementary Figure 4

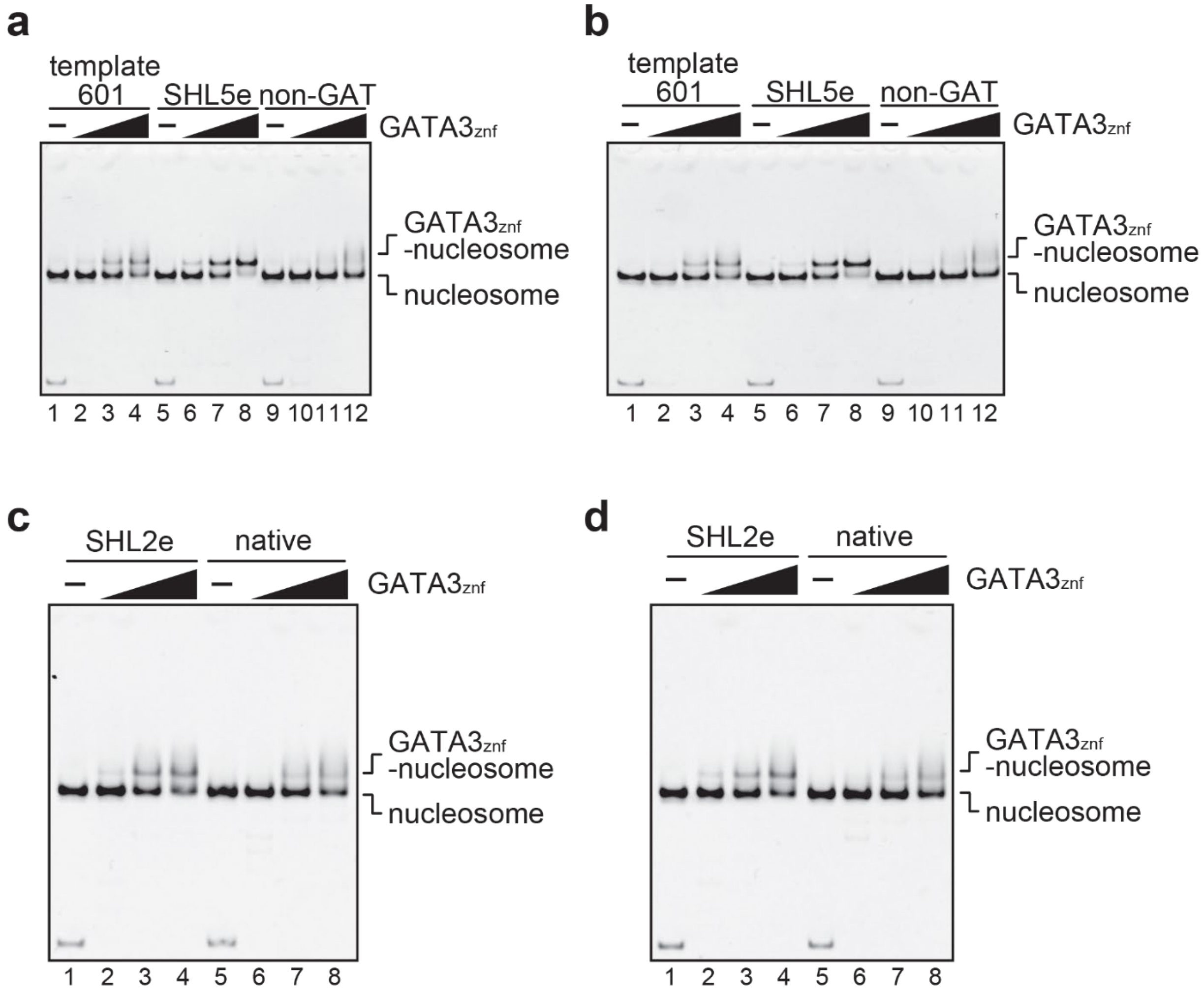

Supplementary Figure 4 Replication of GATA3 nucleosome binding.
a-b. These panels depict independent biological replicates of the experiment also depicted in Figure 3a, making three independent biological replicates. **c-d.** These panels depict independent biological replicates of the experiment depicted in Figure 3c, making three independent biological replicates. The nucleosome (0.33 μM) was mixed with $GATA3_{znf}$ (0 μM, 0.67 μM, 1.0 μM, and 1.33 μM). The reaction mixtures were incubated at 25°C for 30 min and were then analyzed by native-PAGE. The gel was stained with ethidium bromide.

Supplementary Figure 5

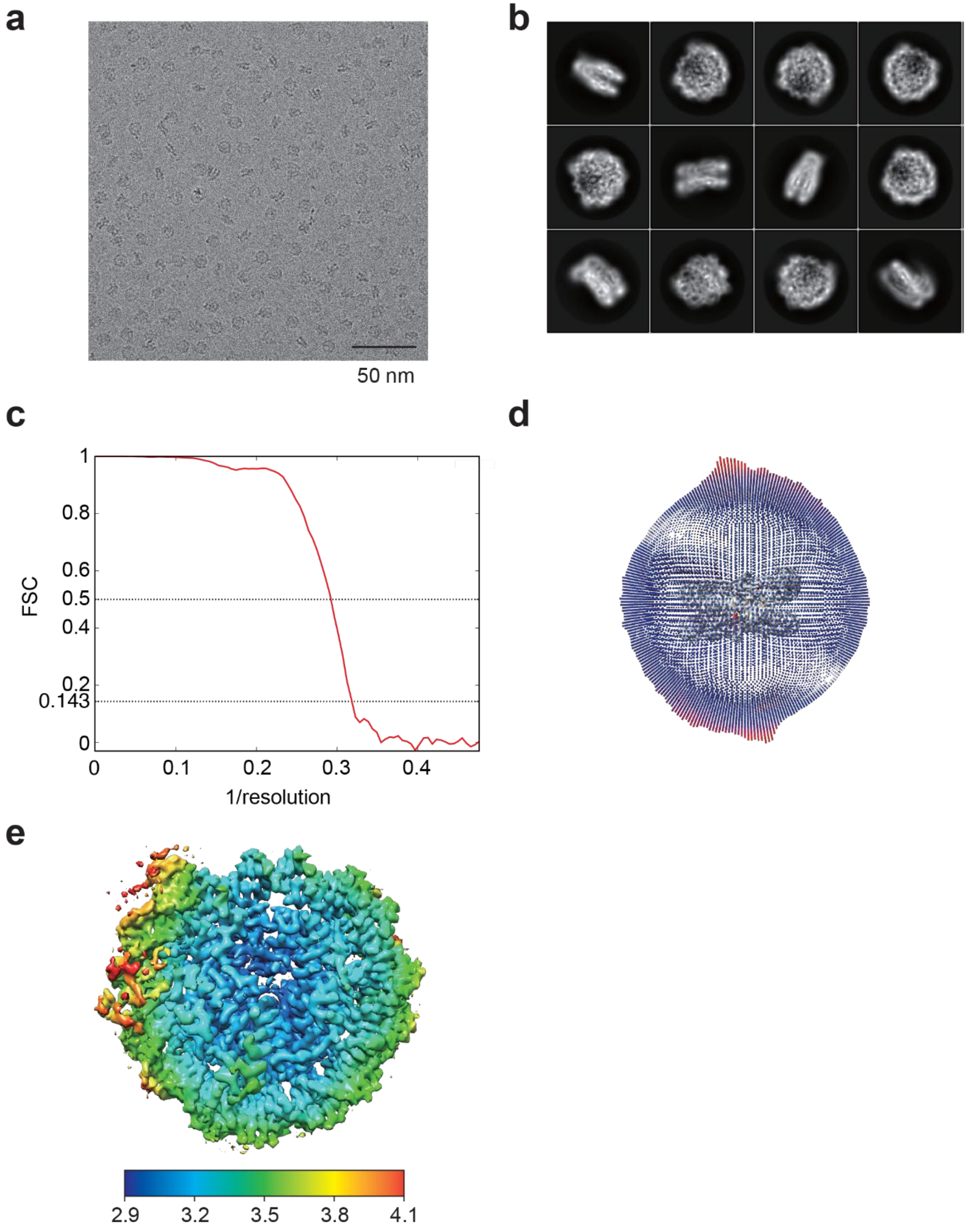

Supplementary Figure 5 Cryo-EM data processing. **a,** Digital micrograph of the $GATA3_{znf}$-nucleosome complex. This experiment was performed using 5610 replicates. Scale bar, 50 nm. **b,** Selected 2D class averages from single particle images of the $GATA3_{znf}$-nucleosome complex. **c,** Fourier Shell Correlation (FSC) curve for the $GATA3_{znf}$-nucleosome complex. The overall resolution of the $GATA3_{znf}$-nucleosome complex is 3.15 Å at FSC = 0.143. **d,** Angular distribution of particle projections of the $GATA3_{znf}$-nucleosome complex. **e,** Local resolution map of the $GATA3_{znf}$-nucleosome complex, showing the resolution across the map from 2.9 Å to 4.1 Å.

Supplementary Figure 6

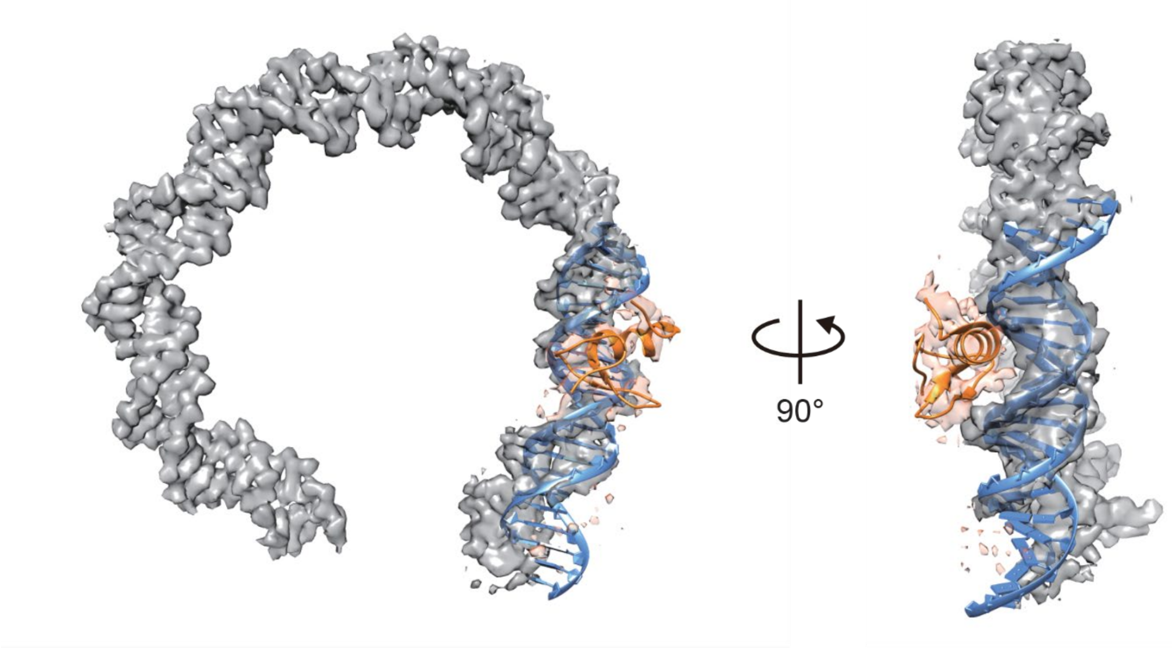

Supplementary Figure 6 Structural comparison between the cryo-EM structure of the $GATA3_{znf}$-nucleosome complex and the crystal structure of the GATA3 zinc finger-DNA complex. The crystal structure of GATA3 zinc finger-DNA complex (PDB ID: 4HC9) was docked into the cryo-EM density map by rigid body fitting in UCSF Chimera. GATA3 zinc finger and DNA crystal structures were colored by orange and light blue, respectively. The cryo-EM density maps correspond to $GATA3_{znf}$ and nucleosomal DNA were colored by orange and gray, respectively.

Supplementary Figure 7

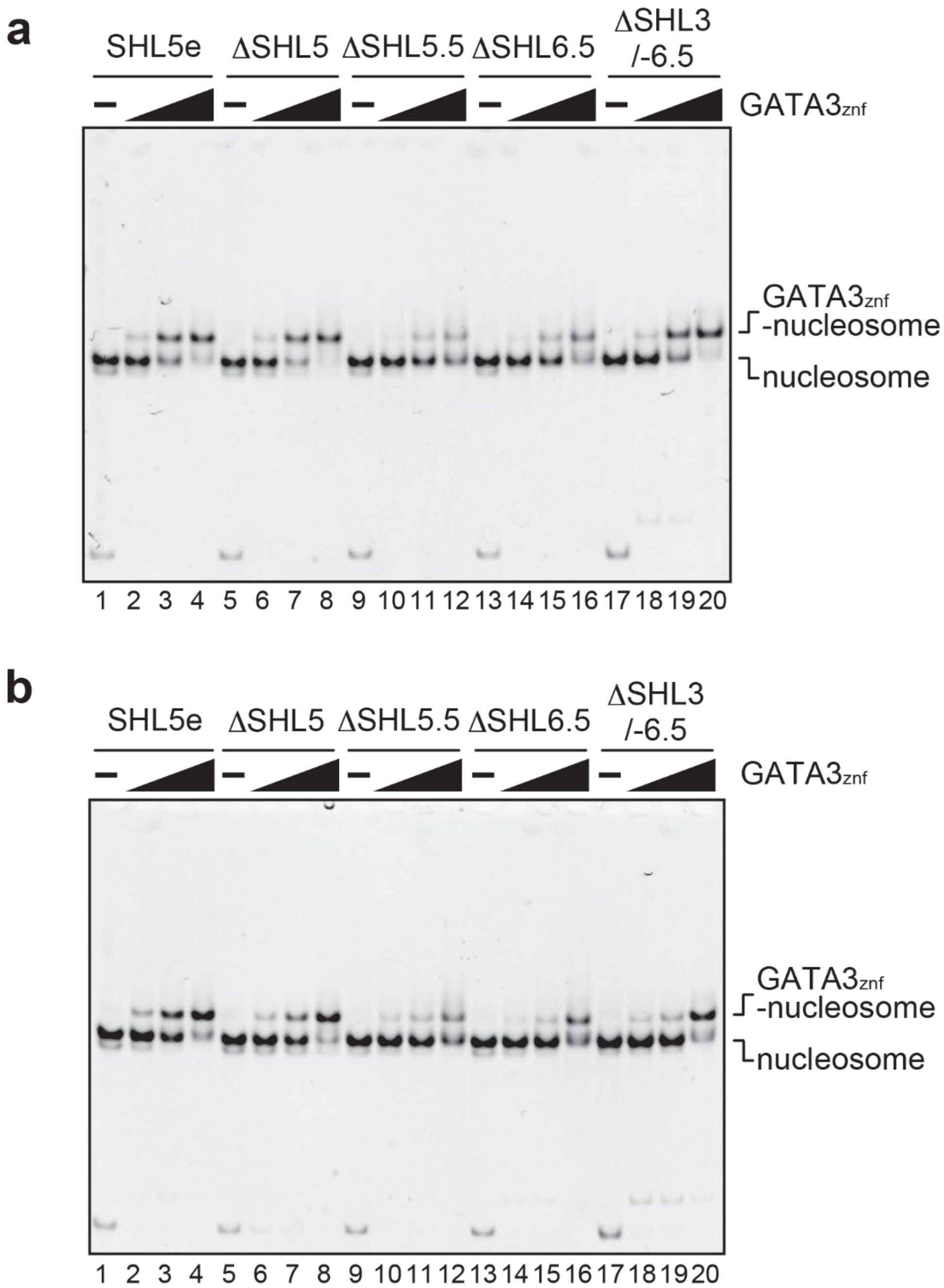

Supplementary Figure 7 Replicated experiments of Fig. 5b. In addition to the experiment presented in Fig. 5b, the assay was independently repeated two times. The nucleosome (0.33 μM) was mixed with GATA3$_{znf}$ (0 μM, 0.67 μM, 1.0 μM, and 1.33 μM). The reaction mixtures were incubated at 25°C for 30 min, and were then analyzed by native-PAGE.

Supplementary Table 1

Cryo-EM data collection statistics

	GATA3-nucleosome (EMDB-0783)
**Data collection and processing**	
Magnification	75,000
Voltage (kV)	300
Electron exposure (e–/Å^2)	50
Defocus range (μm)	1.0-3.0
Pixel size (Å)	1.05
Symmetry imposed	C1
Initial particle images (no.)	1,026,599
Final particle images (no.)	262,357
Map resolution (Å)	3.15
FSC threshold	0.143
Map resolution range (Å)	2.9-4.1

GATNGAT

Total input (number of peaks)		GAT-N0-GAT	GAT-N1-GAT	GAT-N2-GAT	GAT-N3-GAT	GAT-N4-GAT	GAT-N5-GAT	GAT-N6-GAT	GAT-N7-GAT	GAT-N8-GAT	GAT-N9-GAT	GAT-N10-GAT	Total all
11035	G1	2183	1031	2755	2197	2022	2769	2984	2307	2163	3267	2997	26675
19585	G2	3066	1881	3691	3473	2851	4559	5106	3519	3605	5067	4325	41143
11458	G3	1866	1829	2378	2697	1979	3218	4922	2191	2476	3863	2874	30293
43271	random	3986	3291	2910	2893	2852	3839	3266	2914	2528	3312	2810	34601

GATNATC

Total input (number of peaks)		GAT-N0-ATC	GAT-N1-ATC	GAT-N2-ATC	GAT-N3-ATC	GAT-N4-ATC	GAT-N5-ATC	GAT-N6-ATC	GAT-N7-ATC	GAT-N8-ATC	GAT-N9-ATC	GAT-N10-ATC	Total all
11035	G1	727	814	524	3034	2440	705	696	723	721	687	853	11924
19585	G2	955	1012	831	3203	3352	1082	1089	1030	1016	1038	1213	15821
11458	G3	664	635	506	1624	2310	649	663	607	688	778	760	9884
43271	random	1174	1264	1159	1547	1168	1254	1493	1732	1256	1217	1307	14571

GATNGAT

	GAT-N0-GAT	GAT-N1-GAT	GAT-N2-GAT	GAT-N3-GAT	GAT-N4-GAT	GAT-N5-GAT	GAT-N6-GAT	GAT-N7-GAT	GAT-N8-GAT	GAT-N9-GAT	GAT-N10-GAT	Total all
G1	0.197825102	0.093429995	0.249660172	0.199093792	0.183235161	0.250928863	0.270412324	0.209062075	0.196012687	0.296057997	0.271590394	2.417308564
G2	0.156548379	0.09604289	0.188460557	0.177329589	0.14557059	0.232780189	0.260709727	0.179678325	0.184069441	0.258718407	0.22083227	2.100740363
G3	0.162855647	0.159626462	0.207540583	0.235381393	0.172717752	0.280851807	0.42956886	0.191220108	0.216093559	0.337144353	0.250829115	2.643829639
random	0.092117122	0.076055557	0.067250584	0.066857711	0.065910194	0.088719928	0.075477803	0.067343024	0.0584225	0.07654087	0.064939567	0.799634859

GATNATC

	GAT-N0-ATC	GAT-N1-ATC	GAT-N2-ATC	GAT-N3-ATC	GAT-N4-ATC	GAT-N5-ATC	GAT-N6-ATC	GAT-N7-ATC	GAT-N8-ATC	GAT-N9-ATC	GAT-N10-ATC	Total all
G1	0.065881287	0.073765292	0.047485274	0.274943362	0.221114635	0.06388763	0.063072043	0.065518804	0.065337562	0.062256457	0.077299502	1.080561849
G2	0.048761808	0.051672198	0.042430431	0.163543528	0.171151391	0.055246362	0.055603778	0.052591269	0.051876436	0.052999745	0.061935154	0.807812101
G3	0.057950777	0.055419794	0.044161285	0.141735032	0.201605865	0.056641648	0.057863501	0.052976087	0.060045383	0.067900157	0.066329202	0.862628731
random	0.027131335	0.02921125	0.026784683	0.035751427	0.026992674	0.028980148	0.034503478	0.040026808	0.029026369	0.028125072	0.030204987	0.336738231

Supplementary Table 2. Frequencies of GATnGAT and GATnATC motifs

Supplementary table 3. Statistical test of GAT motif frequency at GATA3 peaks

	GATnGATA	GATnATC
GATA3	100950	38541
random1	34575	14461
random2	34473	14508
random3	34370	14635
random4	34864	14997
random5	34270	14598
random6	34574	14723
random7	34017	14844
random8	34309	14657
random9	34229	14478
random10	34400	14700
random11	34261	14679
random12	34706	14666
random13	34286	14458
random14	34360	14542
random15	33718	14704
random16	35655	14867
random17	34476	14571
random18	34322	14628
random19	34925	14746
random20	33961	14564
random21	34191	14781
random22	33849	14728
random23	33750	14584
random24	34320	14737
random25	34404	14666
random26	34476	14877
random27	34157	14491
random28	34210	14680
random29	35292	14729
random30	34690	14718
random31	35059	14585
random32	34085	14533
random33	34353	14722
random34	34333	14556
random35	34276	14618
random36	34978	14852
random37	34590	14689
random38	35019	14953
random39	34456	14770
random40	34476	14760
random41	34606	14889

random42	34376	14717
random43	34849	14765
random44	34556	14843
random45	34371	14574
random46	34536	14858
random47	34739	14568
random48	34464	14659
random49	33145	14718
random50	33973	14653
random51	34281	14733
random52	33848	14680
random53	34997	14583
random54	34986	14576
random55	34695	14493
random56	34724	14689
random57	34033	14759
random58	34615	14743
random59	34305	14672
random60	34856	14909
random61	35098	14840
random62	33993	14571
random63	34173	14850
random64	34648	14634
random65	34571	14627
random66	34544	14825
random67	34640	14546
random68	34389	14647
random69	34041	14533
random70	34361	14667
random71	34344	14884
random72	34594	14708
random73	34486	14740
random74	34402	14587
random75	34722	14771
random76	34360	14690
random77	34669	14675
random78	34598	14783
random79	34469	14797
random80	33928	14383
random81	34301	14710
random82	34775	14719
random83	34181	14759
random84	35011	14511

random85	34363	14815
random86	34763	14745
random87	34474	14667
random88	34069	14723
random89	33872	14467
random90	34389	14808
random91	34591	14630
random92	34046	14479
random93	33876	14624
random94	34101	14293
random95	34378	14633
random96	34625	15079
random97	34006	14703
random98	34295	14794
random99	34042	14784
random100	35016	14911

peak class	N	motif ct	modified Z-score	Chi-square p-value	Bonferroni correction
	0	2183	-0.187	2.55E-07	2.80E-06
	1	1031	-1.921	1.05E-193	1.15E-192
	2	2755	0.675	2.09E-12	2.30E-11
	3	2197	-0.166	1.20E-06	1.32E-05
	4	2022	-0.429	9.23E-18	1.02E-16
G1	5	2769	0.696	2.36E-13	2.60E-12
	6	2984	1.019	1.11E-32	1.22E-31
	7	2307	0	0.01197	0.132
	8	2163	-0.217	2.40E-08	2.64E-07
	9	3267	1.445	6.52E-72	7.17E-71
	10	2997	1.039	3.85E-34	4.24E-33
	0	3066	-0.505	6.33E-31	6.96E-30
	1	1881	-1.615	4.30E-223	4.73E-222
	2	3691	0.081	0.39812	4.379
	3	3473	-0.124	4.57E-06	5.03E-05
	4	2851	-0.706	1.64E-52	1.80E-51
G2	5	4559	0.894	8.80E-45	9.68E-44
	6	5106	1.406	2.60E-121	2.86E-120
	7	3519	-0.081	0.00015	1.63E-03
	8	3605	0	0.02035	0.224
	9	5067	1.370	1.36E-114	1.50E-113
	10	4325	0.675	1.15E-23	1.27E-22
	0	1866	-0.828	1.86E-70	2.05E-69
	1	1829	-0.878	2.72E-76	3.00E-75
	2	2378	-0.133	5.79E-14	6.36E-13
	3	2697	0.300	0.25538	2.809
	4	1979	-0.675	4.23E-54	4.66E-53
G3	5	3218	1.007	1.77E-20	1.95E-19
	6	4922	3.320	0	0
	7	2191	-0.387	2.31E-29	2.54E-28
	8	2476	0	2.79E-08	3.07E-07
	9	3863	1.882	7.30E-109	8.03E-108
	10	2874	0.540	0.01639	0.180

Supplementary table 4. Statistical analysis of differential distribution of GATnGAT motif at each GATA3 peak group

peak class	N	motif ct	modified Z-score	Chi-square p-value	Bonferroni correction
	0	727	0.075	5.74E-30	6.32E-29
	1	814	1.705	7.91E-18	8.70E-17
	2	524	-3.728	3.52E-71	3.87E-70
	3	3034	43.299	0	0
	4	2440	32.170	0	0
G1	5	705	-0.337	1.46E-33	1.61E-32
	6	696	-0.506	4.31E-35	4.74E-34
	7	723	0	1.32E-30	1.46E-29
	8	721	-0.037	6.31E-31	6.94E-30
	9	687	-0.675	1.17E-36	1.29E-35
	10	853	2.436	1.86E-13	2.04E-12
	0	955	-1.098	9.69E-41	1.07E-39
	1	1012	-0.344	4.47E-32	4.91E-31
	2	831	-2.738	2.69E-63	2.96E-62
	3	3203	28.633	0	0
	4	3352	30.604	0	0
G2	5	1082	0.582	6.67E-23	7.33E-22
	6	1089	0.675	4.49E-22	4.94E-21
	7	1030	-0.106	1.46E-29	1.60E-28
	8	1016	-0.291	1.65E-31	1.82E-30
	9	1038	0	1.76E-28	1.94E-27
	10	1213	2.314	4.67E-10	5.13E-09
	0	664	0	2.28E-16	2.51E-15
	1	635	-0.343	2.94E-20	3.24E-19
	2	506	-1.870	6.30E-43	6.93E-42
	3	1624	11.360	3.92E-142	4.32E-141
	4	2310	19.478	0	0
G3	5	649	-0.178	2.52E-18	2.77E-17
	6	663	-0.012	1.70E-16	1.87E-15
	7	607	-0.675	1.97E-24	2.16E-23
	8	688	0.284	1.75E-13	1.92E-12
	9	778	1.349	2.47E-05	2.71E-04
	10	760	1.136	1.25E-06	1.38E-05

Supplementary table 5. Statistical analysis of differential distribution of GATnGAT motif at each GATA3 peak group

Supplementary Table 6. DNA sequences of the nucleosomes used in this work

SHL2a: 5´-
ACTAGAATCCCGGTGCCGAGGCCGCTCAATTGGTCGTAGACAGCTCTAGCACCGCTTAAACG
CACGTACGCGCTGTCCCCCGCGAGATAACATCTAAGGGGATTACTCCCTAGTCTCCAGGCAC
GTGTCACACCTATACATCAGT-3´

SHL2b: 5´-
ACTAGAATCCCGGTGCCGAGGCCGCTCAATTGGTCGTAGACAGCTCTAGCACCGCTTAAACG
CACGTACGCGCTGTCCCCCGCGTTAGATACCATCTGGGGATTACTCCCTAGTCTCCAGGCAC
GTGTCACACCTATACATCAGT-3´

SHL2c: 5´-
ACTAGAATCCCGGTGCCGAGGCCGCTCAATTGGTCGTAGACAGCTCTAGCACCGCTTAAACG
CACGTACGCGCTGTCCCCCGCGTTTTAGATACCATCTGGATTACTCCCTAGTCTCCAGGCACG
TGTCACACCTATACATCAGT-3´

SHL2d: 5´-
ACTAGAATCCCGGTGCCGAGGCCGCTCAATTGGTCGTAGACAGCTCTAGCACCGCTTAAACG
CACGTACGCGCTGTCCCCCGCGTTTTAAAGATAACATCTATTACTCCCTAGTCTCCAGGCACG
TGTCACACCTATACATCAGT-3´

SHL2e: 5´-
ACTAGAATCCCGGTGCCGAGGCCGCTCAATTGGTCGTAGACAGCTCTAGCACCGCTTAAACG
CACGTACGCGCTGTCCCCCGCGTTTTAACCAGATAGCATCTTACTCCCTAGTCTCCAGGCACG
TGTCACACCTATACATCAGT-3´

SHL5a: 5´-
ACTAGAATCCCGGTGCCGAGGCCGCTCAATTGGTCGTAGACAGCTCTAGCACCGCTTAAACG
CACGTACGCGCTGTCCCCCGCGTTTTAACCGCCAAGGGGATTACTCCCTAGTCAGATAGCAT
CTGTCACACCTATACATCAGT-3´

SHL5b: 5´-
ACTAGAATCCCGGTGCCGAGGCCGCTCAATTGGTCGTAGACAGCTCTAGCACCGCTTAAACG
CACGTACGCGCTGTCCCCCGCGTTTTAACCGCCAAGGGGATTACTCCCTAGTCTCAGATAAC
ATCTCACACCTATACATCAGT-3´

SHL5c: 5´-
ACTAGAATCCCGGTGCCGAGGCCGCTCAATTGGTCGTAGACAGCTCTAGCACCGCTTAAACG
CACGTACGCGCTGTCCCCCGCGTTTTAACCGCCAAGGGGATTACTCCCTAGTCTCCAAGATA
GCATCTCACCTATACATCAGT-3´

SHL5d: 5´-
ACTAGAATCCCGGTGCCGAGGCCGCTCAATTGGTCGTAGACAGCTCTAGCACCGCTTAAACG
CACGTACGCGCTGTCCCCCGCGTTTTAACCGCCAAGGGGATTACTCCCTAGTCTCCAGGAGA
TAGCATCTCCTATACATCAGT -3´

SHL5e: 5´-
ACTAGAATCCCGGTGCCGAGGCCGCTCAATTGGTCGTAGACAGCTCTAGCACCGCTTAAACG
CACGTACGCGCTGTCCCCCGCGTTTTAACCGCCAAGGGGATTACTCCCTAGTCTCCAGGCAA
GATACCATCTTATACATCAGT -3´

Template 601 sequence: 5´-
ACTAGAATCCCGGTGCCGAGGCCGCTCAATTGGTCGTAGACAGCTCTAGCACCGCTTAAACG
CACGTACGCGCTGTCCCCCGCGTTTTAACCGCCAAGGGGATTACTCCCTAGTCTCCAGGCAC
GTGTCACACCTATACATCAGT-3´

Non-GAT: 5´-
ACTAGACGACCGGTGCCGAGGCCGCTCAATTGGTCGTAGACAGCTCTAGCACCGCTTAAACG
CACGTACGCGCTGTCCCCCGCGTTTTAACCGCCAAGGGTCGTACTCCCTAGTCTCCAGGCAC
GTGTCACACCTCGCCCGAAGT-3´

SHL5e ΔSHL5 mutant: 5´-
ACTAGAATCCCGGTGCCGAGGCCGCTCAATTGGTCGTAGACAGCTCTAGCACCGCTTAAACG
CACGTACGCGCTGTCCCCCGCGTTTTAACCGCCAAGGGGATTACTCCCTAGTCTCCAGGCAC
GTGTCCATCTTATACATCAGT-3´

SHL5e ΔSHL5.5 mutant: 5´-
ACTAGAATCCCGGTGCCGAGGCCGCTCAATTGGTCGTAGACAGCTCTAGCACCGCTTAAACG
CACGTACGCGCTGTCCCCCGCGTTTTAACCGCCAAGGGGATTACTCCCTAGTCTCCAGGCAA
GATACACACCTATACATCAGT-3´

SHL5e ΔSHL6.5 mutant: 5´-
ACTAGAATCCCGGTGCCGAGGCCGCTCAATTGGTCGTAGACAGCTCTAGCACCGCTTAAACG
CACGTACGCGCTGTCCCCCGCGTTTTAACCGCCAAGGGGATTACTCCCTAGTCTCCAGGCAA
GATACCATCTTATACCGAAGT-3´

SHL5e ΔSHL3/-6.5 mutant: 5´-
ACTAGACGACCGGTGCCGAGGCCGCTCAATTGGTCGTAGACAGCTCTAGCACCGCTTAAACG
CACGTACGCGCTGTCCCCCGCGTTTTAACCGCCAAGGGTCGTACTCCCTAGTCTCCAGGCAA
GATACCATCTTATACATCAGT-3´

Native human genome sequence: 5´-
AAGAGGGCAAGTGCCCCAATCTGATTTTCTTATCAATTGGACCTCTAAGTAAATAGATGAAT
GATGTTTGTTAGCAGGAAGAGATTTCATCTGTTGAATACCACTGACTTAGTGTAAATAAATC
TACGATATATGGCGACATAAG-3´
